# Changes in the Host Gut Microbiota during Parasitization by Parasitic Wasp *Cotesia vestalis*

**DOI:** 10.3390/insects13090760

**Published:** 2022-08-24

**Authors:** Shuaiqi Zhang, Jieling Huang, Qiuping Wang, Minsheng You, Xiaofeng Xia

**Affiliations:** 1 State Key Laboratory of Ecological Pest Control for Fujian and Taiwan Crops, Institute of Applied Ecology, Fujian Agriculture and Forestry University, Fuzhou 350002, China; 2 Ministerial and Provincial Joint Innovation Centre for Safety Production of Cross-Strait Crops, Fujian Agriculture and Forestry University, Fuzhou 350002, China; 3 Joint International Research Laboratory of Ecological Pest Control, Ministry of Education, Fuzhou 350002, China

**Keywords:** host gut microbiota, parasitoids, host-parasite-microbe interactions, host regulation

## Abstract

**Simple Summary:**

*Cotesia vestalis* is a larval endo-parasitoid of the diamondback moth (*Plutella xylostella*), which is a severe pest of cruciferous crops. The function of the gut microbiota of insects has been widely studied. However, it was unclear whether, and how, the gut microbiota of *P. xylostella* responds to its natural enemy, *C. vestalis*. In this study, a time-course experiment was performed to examine changes in the host–microbial community from the start of parasitization to the mature stage of the parasitoid larvae. Our results will provide a framework for studies of host-gut microbiota and parasitic wasp interactions.

**Abstract:**

Parasites attack the host insects and possibly impact the host-gut microbiota, which leads to provision of a suitable host environment for parasites’ development. However, little is known about whether and how the parasitic wasp *Cotesia vestalis* alters the gut microbiota of the host *Plutella xylostella.* In this study, 16S rDNA microbial profiling, combined with a traditional isolation and culture method, were used to assess changes in the bacterial microbiome of parasitized and non-parasitized hosts at different developmental stages of *C. vestalis* larvae. Parasitization affected both the diversity and structure of the host-gut microbiota, with a significant reduction in richness on the sixth day post parasitization (6 DPP) and significant differences in bacterial structure between parasitized and non-parasitized hosts on the third day. The bacterial abundance of host-gut microbiota changed significantly as the parasitization progressed, resulting in alteration of potential functional contribution. Notably, the relative abundance of the predominant family Enterobacteriaceae was significantly decreased on the third day post-parasitization. In addition, the results of traditional isolation and culture of bacteria indicated differences in the bacterial composition between the three DPP and CK3 groups, as with 16S microbial profiling. These findings shed light on the interaction between a parasitic wasp and gut bacteria in the host insect during parasitization.

## 1. Introduction

In insects, the gut microbiota plays a substantial role in the host’s life activities, which include digestion, nitrogen fixation [1], detoxification [2], development [3], pesticide resistance [4], behavior [5], and increasing host defenses against abiotic stress [6] and parasites [7]. Intestinal homeostasis is achieved by maintaining microbial populations at a specific density range to avoid excessive losses or to provide the required contribution to the host insect [8,9]. Therefore, characterization of the diversity and composition of gut microbiota in insects is essential for understanding the biology of the host insects [10].

There is increasing evidence that the diversity of the gut microbiome in host insects is influenced by host–parasite interactions, which provides a new perspective for understanding the co-evolution of host–parasite interactions. For the pathogenic fungus *Beauveria bassiana*–*Dendroctonus valens* association, the evenness, structure, and abundance of the host’s bacterial community are substantially altered by infection with *B. bassiana.* The gut bacterium *Erwinia sp.* accelerates the mortality of the host [11]. For the tapeworm *Hymenolepis diminuta*–*Tenebrio molitor* association, considerable alteration in the host-gut bacteriome and mycobiome are found [12]. Recent studies have investigated the influences of parasitic wasp parasitization on the microbiome of their host insects. Changes in the host-gut microbiota caused by parasitization are observed in the host insects parasitized by the wasp *Cotesia flavipes* [13], *Cotesia glomerat* [14], *Lysiphlebia japonica* [15], and *Habrobracon hebetor* [16]. In contrast, trypanosomatid (*Lotmaria passim*) does not impact the general landscape of the honey bee (*Apis mellifera*)-gut microbiota [17]. Therefore, whether, and how, the parasitoid and host-microbiome interact needs to be analyzed specifically for each species.

For parasitic wasp–host interaction, parasitic wasps lay eggs in the hosts, regulate host physiology, and their larvae coexist with the host-gut microbiome in the host [18,19]. It has been indicated that host endosymbionts might influence host resistance to parasitoid wasps, and this has been mainly studied in aphids. Endosymbionts *Hamiltonella defensa* protect the pea aphid *Acyrthosiphon pisum* against the parasitoid wasp *Aphidius ervi* [20]. Endosymbionts *Regiella insecticola* provide vital protection for peach aphids *Myzus persicae* against wasps *Aphidius colemani* [21]. Meanwhile, a study showed that the differences in bacterial communities of *Drosophila melanogaster* influenced its resistance to parasitoids [22]. Furthermore, parasitic wasp embryos [23] and larvae [24,25] rely on nutrients from their hosts for development, and they regulate the metabolism of proteins, carbohydrates, and lipids in their hosts to satisfy their nutritional demands [26,27,28]. Besides these points, parasitic wasps modulate the host’s immune system during adaptation, and studies have shown that they may suppress the expression of host antimicrobial peptide genes and Toll and IMD immune pathways [29,30,31], all of which are known to be important for maintaining host-gut microbial homeostasis [32,33,34]. These results indicate that parasitic wasps may influence the host microbiota by regulating host immunity. Collectively, the host microbiome and parasitic wasps are likely to interact. 

The diamondback moth, *Plutella xylostella*, an important pest of cruciferous vegetable crops, causes severe economic losses worldwide [35]. The parasitic wasp, *Cotesia vestalis*, is a solitary endophagous parasitoid of *P. xylostella* larvae [36,37]. Several studies have investigated that the gut microbial diversity and composition of *P. xylostella* varied according to food type [38], insecticidal protoxins [39], insecticide resistance [40], and antibiotics [41]. However, changes in the gut microbiota of *P. xylostella* due to parasitism remain poorly understood. To explore whether the host-gut microbiota is involved in the interaction between host and parasite, 16S rDNA sequencing and traditional isolation and culture methods were performed to study the changes in the diversity and potential functions of gut microbiota in *P. xylostella* larvae when parasitized by *C. vestalis*. Our findings serve as a foundation for further studies into the association between the host-gut microbiota and parasitic wasps. 

## 2. Materials and Methods 

### 2.1. Insect Rearing and Sample Collection

Both *P. xylostella* and *C. vestalis* were initially collected from a cabbage-planting field in Fuzhou, China (25.95° N, 119.27° E) in May 2014. Then *P. xylostella* was reared on radish while *C. vestalis* was reared on the larvae of *P. xylostella*. Both insects and radishes were kept under controlled conditions (25 ± 2 °C, 60% ± 10% relative humidity, and 14 light:10 dark photoperiod) in the laboratory.

**Figure 1:**
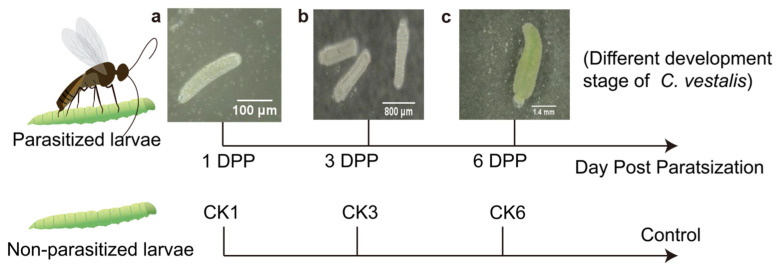
Experimental outline for exploring changes in the gut microbiota of parasitized *P. xylostella* and non-parasitized *P. xylostella*. (Different development stages of *C. vestalis* during sampling. (**a**) egg; (**b**) low instar larva; (**c**) mature larvae).

All samples were divided into two groups: parasitized larvae and non-parasitized larvae. The late second instar larvae of *P. xylostella* were individually exposed to mated *C. vestalis* for parasitization to collect parasitized *P. xylostella*. The control groups were left unparasitized. For 16S rDNA sequencing, samples from parasitized larvae were collected on the first (1 DPP), third (3 DPP), and sixth day post-parasitization (6 DPP) according to the different development stages of the parasitic wasp [42]. Non-parasitized larvae were selected at the instar consistent with the parasitized larvae due to parasitized *P. xylostella* growing slower than non-parasitized ones (Figure 1). In addition, the samples of 3 DPP (the third day post-parasitization) and CK3 (unparasitized control group 3) were collected for the traditional isolation and culture of the gut bacteria. All larvae were soaked in 75% ethanol for 90 s and rinsed in sterile water three times. Then the surface-sterilized *P. xylostella* larvae were dissected in sterile 1% phosphate-buffered saline (PBS) under a microscope. For parasitized larvae, the gut of *P. xylostella* was collected when the eggs and larvae of *C. vestalis* were observed under a microscope. Eventually, each gut sample was stored at −80 °C until used. Four biological replicates per treatment were collected. Each biological replicate contained guts from 30 *P. xylostella* larvae.

### 2.2. DNA Extraction and PCR Amplification of 16S rDNA Sequencing

Bacterial genomic DNA was extracted from the 24 gut samples using the E.Z.N.A.^®^ soil DNA Kit (Omega Bio-Tek, Norcross, GA, USA). The 16S rRNA gene hypervariable region V3-V4 was amplified with primer set 338F/806R (Table S1) [43,44]. The PCR reaction was performed in a 20 μL volume including 4 μL 5 × Fast Pfu buffer, 2 μL 2.5 mM dNTPs, 0.8 μL each primer (5 μM), 0.4 μL Fast Pfu polymerase, 10 ng of template DNA, and appropriate ddH_2_O. Cycling conditions were at 95 °C for 3 min, followed by 27 cycles at 95 °C for 30 s, 55 °C for 30 s, and 72 °C for 45 s, with a single extension at 72 °C for 10 min. All samples were amplified in triplicate. The PCR product obtained was purified using the AxyPrep DNA Gel Extraction Kit (Axygen Biosciences, Union City, CA, USA) and quantified using Quantus™ Fluorometer (Promega, Madison, WI, USA). Purified amplicons were paired-end sequenced on an Illumina MiSeq PE300 platform (Illumina, San Diego, CA, USA). The double-ended raw sequences were quality-filtered using fastp [45] and merged using FLASH [46], according to the following: (i) Reads of 300 bp were truncated at any site with an average quality score < 20 over a sliding window of 50 bp. Only reads ≥ 50 bp were retained. Reads containing N bases were removed. (ii) Overlapping sequences longer than 10 bp were assembled in which the maximum mismatch ratio was 0.2. Only assembled reads were used for the following analysis. (iii) Samples were distinguished according to the barcode (exact matching) and primers (2 nucleotide mismatch in matching). 

Unique read sequences were identified from the optimized sequences (dereplication), singletons were discarded, and, then, these sequences were clustered into operational taxonomic units (OTUs) using UPARSE 7.1 at a 97% sequence similarity level [47]. Chimeras were removed during clustering. Chloroplast and mitochondrion sequences were removed for further analysis. The ribosomal database project (RDP) classifier (Version 2.11) was used to identify taxonomic groups based on the e SILVA 16S rRNA database [48] using a confidence threshold of 80% [49,50]. The raw data were submitted to the NCBI Sequence Read Archive (SRA) database (Accession Number: SAMN28027321- SAMN28027344). 

### 2.3. Sequence Data Analysis

Based on the rarefied OTUs, rarefaction curves and alpha diversity indices were calculated with Mothur v1.30.1, including the observed richness (Sobs) and Shannon index [51]. The principal coordinate analysis (PCoA) based on Bray-Curtis dissimilarity was applied to determine the compositional difference of microbial communities, with ANOSIM (1000 permutations) testing the significance of the difference between samples. PICRUSt2 (Phylogenetic Investigations of Communities by Reconstruction of Unobserved States) was a bioinformatic tool for predicting and comparing functional attributes of microbial communities [52,53,54,55,56,57]. The potential function prediction of host-gut microbiota was analyzed by PICRUSt2 based on OTU representative sequences and abundances. All comparisons between two groups were analyzed by the Wilcoxon rank-sum test using Stats Package (R, version 3.3.1). 

### 2.4. Isolation of Host Gut Bacteria 

Thirty-five larvae from the 3 DPP and CK3 groups were randomly selected. The guts of surface-sterilized worms were separated and homogenized in sterile centrifuge tubes containing 1 mL 1% PBS solution. Ten-fold serial (10^−1^, 10^−2^, 10^−3^, 10^−4^, and 10^−5^) dilutions of homogenized suspension were plated on four media, including Bile Aesculin Azide Agar (selective media for *Enterococcus*), Salmonella-Shigella Agar (selective media for *Salmonella*), Nutrient Agar (general media for bacteria), and Luria Bertani (general media for bacteria), and subsequently incubated at 37 °C. Plates were observed every 12 h to obtain the original bacterial strains. The isolates were categorized according to differences in colony size, color, and morphology. Then distinct morphological colonies were purified on LB plates for at least five generations to obtain monoclonal strains, followed by storing in 50% glycerol at −80 °C. The bacterial isolates obtained were grown in 500 µL liquid LB medium at 37 °C for 2–3 h. The 16S rRNA sequence was amplified by using universal primers 27F/1492R (Table S1) and the bacterial culture as a template. The PCR product was blasted in the NCBI database after sequencing. The 16S rRNA sequences of the bacteria isolated were deposited in the NCBI GenBank database with the accession number presented in Table S2. Furthermore, for evaluating the evolutionary relationships of all bacterial isolates and their closely related species, the phylogenetic tree was constructed by neighbor-joining analysis using MEGA 11.0 software [58]. 

## 3. Results

### 3.1. Effects of Parasitization on Host Gut Microbial Community Diversity and Structure by C. Vestalis

**Figure 2:**
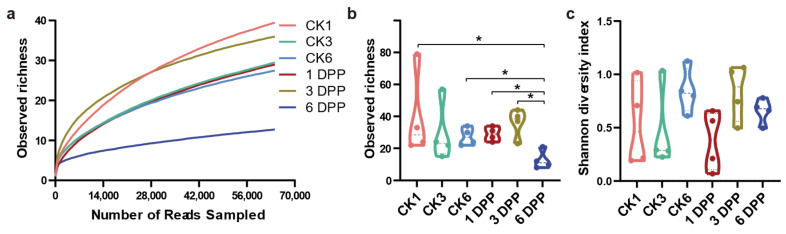
Alpha diversity of the host gut microbiome in the parasitized (CK1, CK3, CK6) and non-parasitized (1 DPP, 3 DPP, 6 DPP) groups at the OTU level. (**a**) Rarefaction curves based on Sobs values (the observed richness); (**b**,**c**) Violin plot showing sobs and Shannon values of bacterial communities in different samples. Wilcoxon rank-sum test between two independent samples was performed among treatments. The symbol “*” indicates statistically significant differences between the two groups being compared (*p* < 0.05).

The 16S rDNA gene hypervariable region V3-V4 was sequenced in 24 samples of parasitized and non-parasitized *P. xylostella*, which yielded 2,173,198 sequences after standard quality filtering. The average length of the reads obtained from all samples was 428 bp. The sequences were clustered into 156 OTUs at 97% sequence identity using rarefied reads (64,327 reads per sample) for 1, 3, and 6 days post-parasitization (DPP), as well as for the control group. The rarefaction curves in all samples indicated adequate sampling and successful retrieval of OTUs. Rarefaction curves of all samples were flattened, showing that the actual bacterial diversity was effectively covered by sequencing (Figure 2a). 

**Figure 3:**
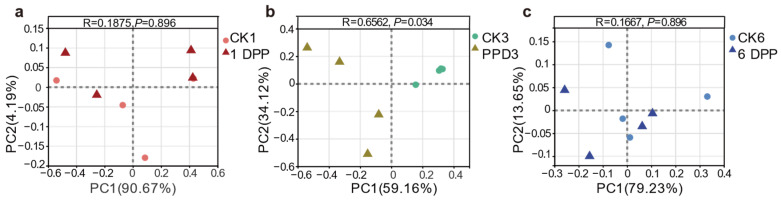
Principle coordinate analysis (PCoA) of the rarefied OTUs comparing the gut microbiota between parasitized and naïve control *P. xylostella* in different time categories with Bray-Curtis dissimilarity distance. (**a**) CK1 vs. 1 DPP, (**b**) CK3 vs. 3 DPP, (**c**) CK3 vs. 3 DPP. Analysis of similarities (ANOSIM) analyses revealed that the samples at 3 DPP were substantially different from those in the CK3 group (*p* = 0.034).

The bacterial community diversity and structure of parasitized and non-parasitized *P. xylostella* were analyzed using alpha diversity and beta diversity, respectively. The sobs index, reflecting microbial community richness, was significantly reduced on the 6 PPD compared with the other two parasitized groups (1 PPD and 3 PPD) (Wilcoxon rank-sum test, *p* = 0.03038). However, this difference at different developmental stages was not observed in the non-parasitized groups. Moreover, 6 PPD had a significantly lower value for the sobs index than the CK6 samples (*p* = 0.03038) (Figure 2b). In all time categories, however, there were no significant differences in community diversity evaluated by the Shannon index between parasitized and non-parasitized *P. xylostella* gut samples (Figure 2c). Taken together, the parasitization by *C. vestalis* decreased host bacterial community richness relative to that of non-parasitized *P. xylostella* on the sixth day after parasitization. Principle coordinate analysis (PCoA) of Bray-Curtis distances showed an apparent separation between the parasitized and control larvae on the third day after parasitization (ANOSIM, *p* = 0.034) (Figure 3b). By contrast, 1 and 6 DPP clustered closely with their respective controls (Figure 3a,c). In conclusion, the changes in gut bacterial structure between parasitized and control hosts were more apparent on the third day after parasitism than at the other two development times.

### 3.2. Impact of Parasitization on the Composition of Host-Gut Microbiota 

**Figure 4:**
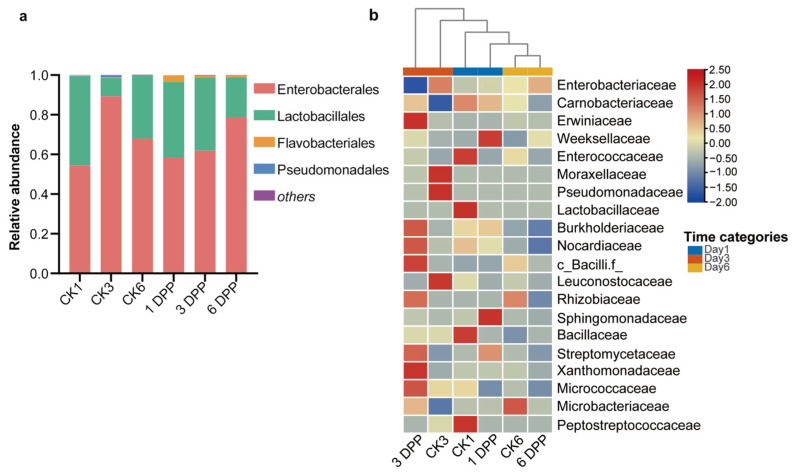
Impact of parasitization on the composition of host-gut bacterial community. (**a**) Relative abundance in the host-gut microbiome at the order level. “Others” included < 1% relative abundance taxa. (**b**) Heatmap of the family abundance in the *P. xylostella* gut microbiome in different time categories. Columns were clustered using the average method based on Euclidean distance, and rows were normalized.

Taxonomic analysis revealed that the major bacteria at the phylum level in all samples were Proteobacteria, Firmicutes, and Bacteroidetes, but these phyla did not significantly change between parasitized *P. xylostella* and their respective control groups (Tables S3 and S4). The host-gut bacterial community was dominated by four bacterial orders: Enterobacteriales, Lactobacillales, Pseudomonadales, and Flavobacteriales (Figure 4a). Among them, the proportion of Pseudomonadales was significantly reduced on 6 DPP compared to CK6 (Wilcoxon rank-sum test, *p* = 0.02107) (Table S4).

**Figure 5:**
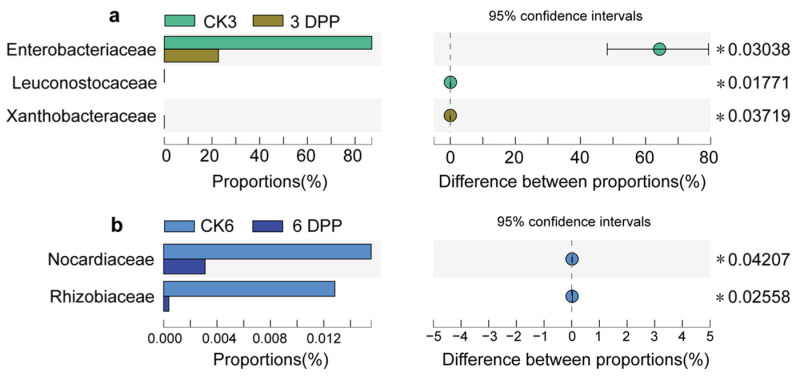
The difference in relative proportion (%) between parasitized and non-parasitized larvae at different sampling times at the family level. (**a**) CK3 vs. 3 DPP, (**b**) CK6 vs. 6 DPP. Statistical analysis was performed by the Wilcoxon rank-sum test. The symbol * indicates *p* < 0.05.

A heatmap was plotted with the relative abundance of the top 20 shared families in six groups. The clustering of the gut samples at the family level indicated that the 3 DPP group showed dissimilarity from the other groups (Figure 4b). Among the top 20 families, in terms of abundance, the abundance of Enterobacteriaceae (*p* = 0.03038) and Leuconostocaceae (*p* = 0.01771) on the 3 DPP showed lower proportions compared with the CK3 group, whereas Xanthobacteraceae in 3 DPP was significantly more abundant than the control (*p* = 0.03719) (Figure 5a). The abundance of Nocardiaceae (*p* = 0.04207) and Rhizobiaceae (*p* = 0.02558) decreased on the 6 DPP compared with CK6 group (Figure 5b).

At the genus level, the gut bacterial community was dominated by *Enterobacter*, *Carnobacterium*, *Pantoea,* an unidentified genus of Enterobacteriaceae, and *Chryseobacterium*, with at least 1% relative abundance (Figure S1). Alterations in bacterial proportions were seen at the genus level, which was consistent with the family level. In particular, significant reductions in the genus *Enterobacter* were observed on the 3 DPP compared with non-parasitized larvae (Figure S2) (*p* = 0.03038). *Pantoea* was one of the dominant bacteria enriched mainly in 3 DPP with a mean relative abundance of 39.29% (Table S3). However, no significant change in the bacterial proportions was observed on the third day after parasitization compared to the control, as one replicate of the 3 DPP sample had lower values than the others (Table S4).

### 3.3. Effects of Parasitization on Host-Gut Microbial Function by C. vestalis

**Figure 6:**
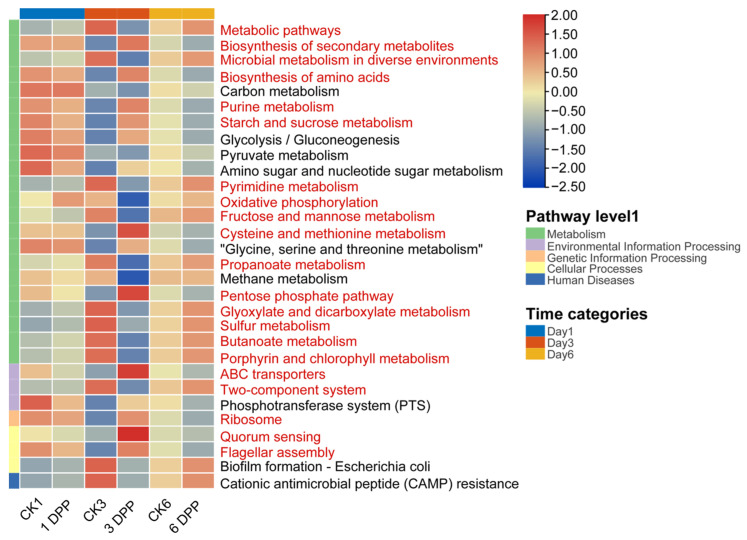
Relative abundance (%) of host-gut microbiota functions between parasitized and non-parasitized larvae at different time categories at the Kyoto Encyclopedia of Genes and Genomes (KEGG) level 3. The heatmap plot was normalized by row. The red letter indicates that the special function at KEGG pathway level 3 significantly differed from the control group during the parasitism (*p* < 0.05). Group color bars on the left indicate that the functions were grouped according to pathway level 1.

The different functional contribution of host-gut microbiota was predicted using the top thirty shared Kyoto Encyclopedia of Genes and Genomes (KEGG) level 3 inferred by PICRUSt2 in all samples. The roles of parasitized and non-parasitized host-gut microbes mostly comprised Metabolism, Genetic Information Processing, Environmental Information Processing, Cellular Processes, and Human diseases. In the most prevalent metabolism category, pathways related to the biosynthesis of secondary metabolites, biosynthesis of amino acids, pentose phosphate pathway, purine/starch and sucrose/cysteine and methionine metabolism predominated on 3 DPP. In contrast, metabolic pathways, microbial metabolism in diverse environments, oxidative phosphorylation, pyrimidine/fructose and mannose/propanoate/glyoxylate and dicarboxylate/butanoate/sulfur/porphyrin and chlorophyll metabolism were significantly reduced. In other functional categories, ABC transporters, ribosome, quorum sensing and flagellar assembly were increased significantly in the 3 DPP group, while the two-component system was more predominant in CK3. However, no significant difference was observed between parasitized and non-parasitized hosts on the first and sixth days (Table S5). Above all, the 3 DPP group showed the most obvious changes in the relative abundance of bacterial functions compared to CK3 among all-time categories, similar to the differences in the structure and composition of host-gut microbiota (Figure 6).

### 3.4. Isolation and Culture of Bacteria from Parasitized and Non-Parasitized Host Gut

As indicated in the high throughput sequencing results, the beta-diversity, composition, and specific function of host-gut bacteria were more variable than the other two development stages on the 3rd day post-parasitization compared to control. According to these changes, the gut samples from 3 DPP and CK3 were chosen to explore the difference in gut microbiota between parasitized and non-parasitized *P. xylostella* using the traditional isolation and culture methods. 

**Figure 7:**
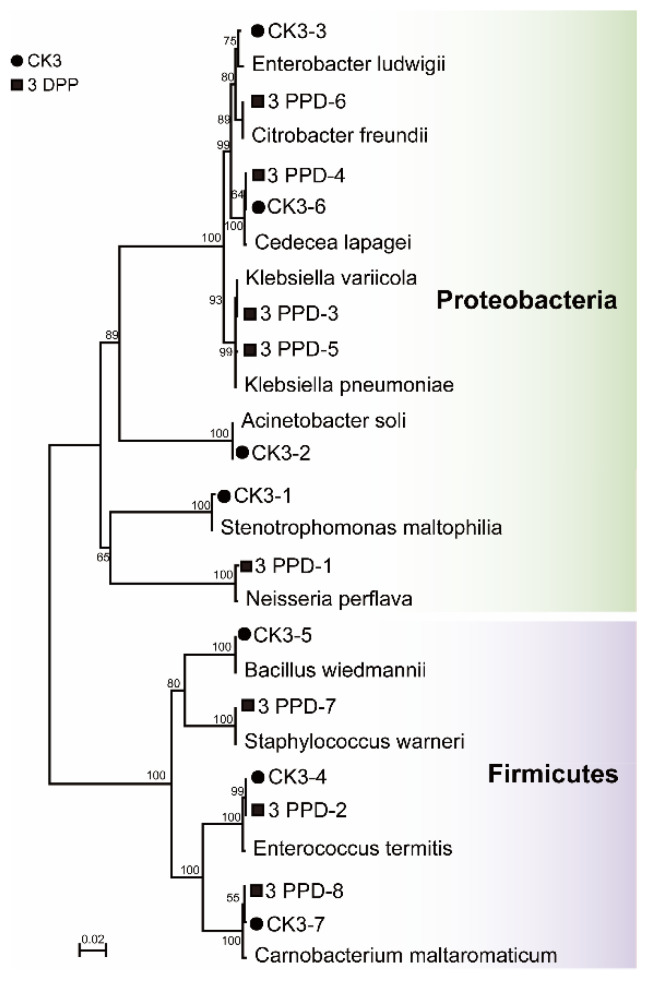
Neighbor-joining tree of bacterial isolates from parasitized and non-parasitized *P. xylostella* and their closely related species based on sequencing of the 16S rDNA gene. The nodes’ bootstrap values were based on 1000 replicates. The scaled bar represents 0.02 estimated phylogenetic divergence.

The 16S rDNA gene sequencing analysis resulted in the identification of 7 species from 3 DPP and 8 species from CK3. The bacterial isolates identified as *Cedecea lapagei* (CK3-6, 3 DPP-4), *Carnobacterium maltaromaticum* (CK3-7, 3 DPP-8), and *Enterococcus termitis* (CK3-4, 3 DPP-2) were present in both groups. Four bacterial isolates from the genera *Stenotrophomonas* (CK3-1), *Acinetobacter* (CK3-2), *Enterobacter* (CK3-3), and *Bacillus* (CK3-5) were uniquely found in the unparasitized control group. Moreover, there were five strains specific to the 3 DPP group, containing the genus *Neisseria* (3 DPP-1), *Klebsiella* (3 DPP-3, 3 DPP-5), *Citrobacter* (3 DPP-6), and *Staphylococcus* (3 DPP-7) (Table S2). Phylogenetic analysis of all isolates with the closest relatives showed that the prevalent phyla were Proteobacteria and Firmicutes in both groups, consistent with the high throughput sequencing results (Figure 7).

## 4. Discussion

How the gut microbiota of *P. xylostella* change due to parasitization by *C. vestalis* at different development stages was investigated in this work. In terms of alpha diversity, we discovered that the bacterial community richness index (sobs) decreased in the late stage of parasitization (6 DPP), whether compared to the early phase of the parasitization process or the non-parasitized group. Interestingly, all the microbial diversity in aphids (*Aphis gosypii*) parasitized by *Lysiphlebia japonica* was lower than that in non-parasitized aphids at 8 h, 16 h, 1 day, 2 days, and 3 days [15]. Additionally, rare microbial taxa have been proven to contribute to community stability and persistence [59,60], so we retained the low-abundance OTUs. The existence of low-abundance OTUs in the other groups was most likely responsible for the Sobs index decreasing in the 6 DPP group and the Shannon value remaining similar to that of the other groups. However, the beta diversity showed that the gut bacterial structure of the host altered significantly compared with the control only on the third day. As previously demonstrated for *C. flavipes*, whereas alpha-diversity analysis revealed changes in the richness of gut microbiota at different stages (1, 5, and 9 “days after parasitization, DAP”) of *D. saccharalis* parasitization by *C. flavipes*, the beta-diversity analysis revealed that the parasitoid influenced the host-gut microbiota only on 5 DAP [13]. The findings suggest that the response mode of host-gut microbiota to parasitoid varies at different phases of parasitization. It has been shown that the nutritional physiology [61] and immune response capacity [62] of the host are different at various stages of parasitoid larval development and may influence the dynamics of microbial diversity in the host.

According to the taxonomic analysis, the bacterial microbiome of non-parasitized *P. xylostella* was dominated by Enterobacteriaceae, followed by Carnobacteriaceae. A previous study has also shown that these two families are the most abundant in the gut of *P. xylostella* [63]. However, significant declines in Enterobacteriaceae of samples at 3 DPP were reported in our investigation, resulting in the Enterobacteriaceae no longer being the most abundant family in the bacterial microbiome on the third day post-parasitization. A previous study has also observed that parasitoid envenomation led to a predominant shift of gut bacterial composition in *Galleria mellonella* [16]. This suggests that *C. vestalis* may significantly disturb the composition of host-gut microbiota in the middle phase of parasitization. The declines in Enterobacteriaceae appear to have been caused by the genus *Enterobacter*, with a similar change in proportions at the genus level. The *Enterobacter* sp. isolated from the gut of *Bactrocera oleae* significantly reduced parasitism rate and fecundity of *Diachasmimorpha longicaudata*. This suggests that the reduction of *Enterobacter* from the *P. xylostella* gut may impact the suitability of the host environment for the *C. vestalis*. Furthermore, the abundance of Enterobacteriaceae recovered to the highest family on 6 DPP, while PCoA analyses showed a similar bacterial structure to CK6, reflecting that the greater impact of parasitic wasps on the microbial community in the host at the 3 DPP was temporary. According to a recent study, the total count of hemocytes in *Diatraea saccharalis* was lowest on the third day after parasitization by *Cotesia flavipes*, while hemocyte viability was significantly higher at 5 DAP for parasitized larvae compared with non-parasitized larvae over 0–10 DAP [64]. The dynamic of the host bacterial community in our study may be due to the immune regulation of the host insect by the parasitic wasp during its development. Previously, researchers considered that alternations in the structure of the gut microbiome could contribute to the variations in the susceptibility to pathogenic microorganisms [65]. Alterations in the host-gut microbiome generated by parasitoid envenomation were found to enhance fungal infection [16]. Whether the interaction between *P. xylostella* and *C. vestalis* leads to similar results remains to be further studied.

It is worth noting that, due to the reduced relative abundance of *Pantoea* in one sample of 3DPP compared to the other replicates, the difference in the abundance of *Pantoea* between 3DPP and CK3 was not significant. Nevertheless, *Pantoea* became the most dominant genus in the host-gut microbiome on the third day after parasitization. *Pantoea* strains are commonly found in the guts of insects [66]. *Pantoea agglomerans* was previously found to produce antifungal phenols, which may play a role in host defense and have an important impact on the composition of the gut flora [67]. Based on its high abundance in 3DPP, it is worth continuing to pay attention to the changes and functions of this kind of flora in future studies. 

The unique structure and physicochemical environment of the insect gut result in a complex and functionally diverse gut microbial community [1]. In the current study, the main functional groups of gut microbiota in parasitized and non-parasitized larvae were similar, and it is assumed that fixed groups play a role in the host, which may be the result of their co-evolution with the host. Additionally, functional KEGG pathway analysis revealed significant differences between samples from the 3 DDP group and CK3, with specific pathways increasing or decreasing in relative abundance. A previous report also suggested that *C. flavipes* might alter the potential function of its host-gut microbiota [13]. Significant differences in gut microbiota functional profiles between parasitized and non-parasitized hosts were mainly enriched to several metabolism-related pathways. These differences suggest that the gut bacteria may affect nutrient replenishment and food digestion in the parasitized *P. xylostella*. Previous studies found that parasitic wasps could regulate the host’s metabolic levels to provide a suitable environment for the development of wasps [68,69], and gut microbiota may play a role in this regulation. However, considering the limitations of PICRUSt2, the analysis to predict the function of gut microbiota only provided some preliminary results. Based on these results, the functional shifts of the host-gut microbiota during parasitization might be determined by combining metabonomics and metagenomics in the future. In addition, hosts in the mid-stage of parasitization could be chosen as study objects in future experiments. 

In this study, Proteobacteria and Firmicutes were the most common phyla that could be cultured in *P. xylostella*. A previous investigation also found that cultured bacterial strains isolated from *P. xylostella* were dominated by these two phyla [70]. Furthermore, the results of traditional isolation and culture of bacteria also indicated differences in the host-gut bacteriome during parasitization. The original strains isolated from *P. xylostella* gut provide valuable resources for the future study of their functions in the interaction between *P. xylostella* and *C. vestalis.* Besides, the bacterial isolates from genus *Neisseria*, *Klebsiella*, and *Citrobacter* obtained using culture methods on the 3 DPP were not detected by high-throughput sequencing, which may be due to the methodological nature of OTU picking and the limitations of taxonomic databases inserting important biases in community analyses. There were still many limitations in this study. Our selection of media types is not yet comprehensive, and the culture was only conducted in an aerobic environment. Further exploration of the culturable bacteria in *P. xylostella*, with a broader range of media and culture methods, is still required.

## 5. Conclusions

To the best of our knowledge, this study provides the first comprehensive description of shifts in the gut bacteriome of *P. xylostella* during parasitization by *C. vestalis*. The degree of changes in bacterial community structure and composition caused by *C. vestalis* varied at the different larval developmental stages of wasps according to the time-series experiments. The most obvious alterations in the structure and composition of host-gut microbiota at 3 DPP affect the potential functional contribution of the gut bacterial community. These alterations suggest that *C. vestalis* larvae may adapt and regulate their host environment by changing the balance of host-gut microbiota. However, the specific biological significance of bacteria cultured from parasitized *P. xylostella*, as well as the mechanisms causing changes in the host microbial community, remain to be tested. In conclusion, our results provide a framework of interactions among *P. xylostella*, its symbionts, and its parasitic enemy, *C. vestalis*, wherein regulation of the host by the parasitic wasp is associated with host-gut bacteria, which could help in understanding the regulation of host by parasitic wasp associated with host-gut bacteria.

## Data Availability

Not applicable.

## Supplementary Materials

The following supporting information can be downloaded at: https://www.mdpi.com/article/10.3390/insects13090760/s1, Table S1: Primer sequences for this study; Table S2: Online blast-based alignment of 16S rDNA gene for cultured gut bacteria; Table S3: Relative abundance in the host-gut microbiome at the phylum, order, family, and genus level; Table S4: Comparison of relative abundance of host-gut microbiota at the phylum, order, family, and genus level between parasitized and non-parasitized groups; Table S5: Comparison of relative abundance of host-gut microbiota function at KEGG pathway level 3 with significant differences between parasitized and control groups; Figure S1: Relative abundance in the host-gut microbiome at the genus level; Figure S2: The difference in relative proportion (%) between parasitized and non-parasitized larvae at different sampling times at genus level.
