# Supplementary material for: Changes in the Host Gut Microbiota during Parasitization by Parasitic Wasp Cotesia vestalis
Source: Insects. 2022 Aug 24;13(9):760. doi: 10.3390/insects13090760 (PMC9506224; doi:10.3390/insects13090760)
Supplement: Supplementary file 1 [file insects-13-00760-s001.zip › insects-1869976-Figure S1.pdf]

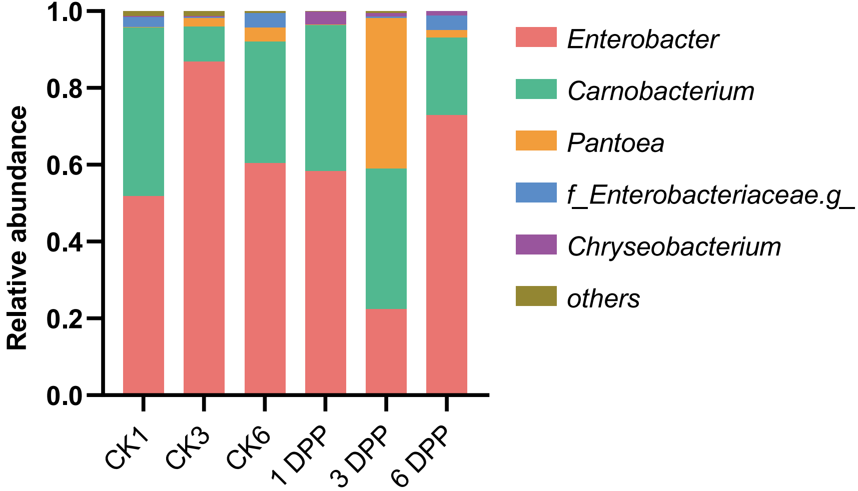

Figure S1. Relative abundance in the host gut microbiome at the genus level. “Others” included < 1% relative abundance taxa.
