# Supplementary material for: Changes in the Host Gut Microbiota during Parasitization by Parasitic Wasp Cotesia vestalis
Source: Insects. 2022 Aug 24;13(9):760. doi: 10.3390/insects13090760 (PMC9506224; doi:10.3390/insects13090760)
Supplement: Supplementary file 1 [file insects-13-00760-s001.zip › insects-1869976-Figure S2.pdf]

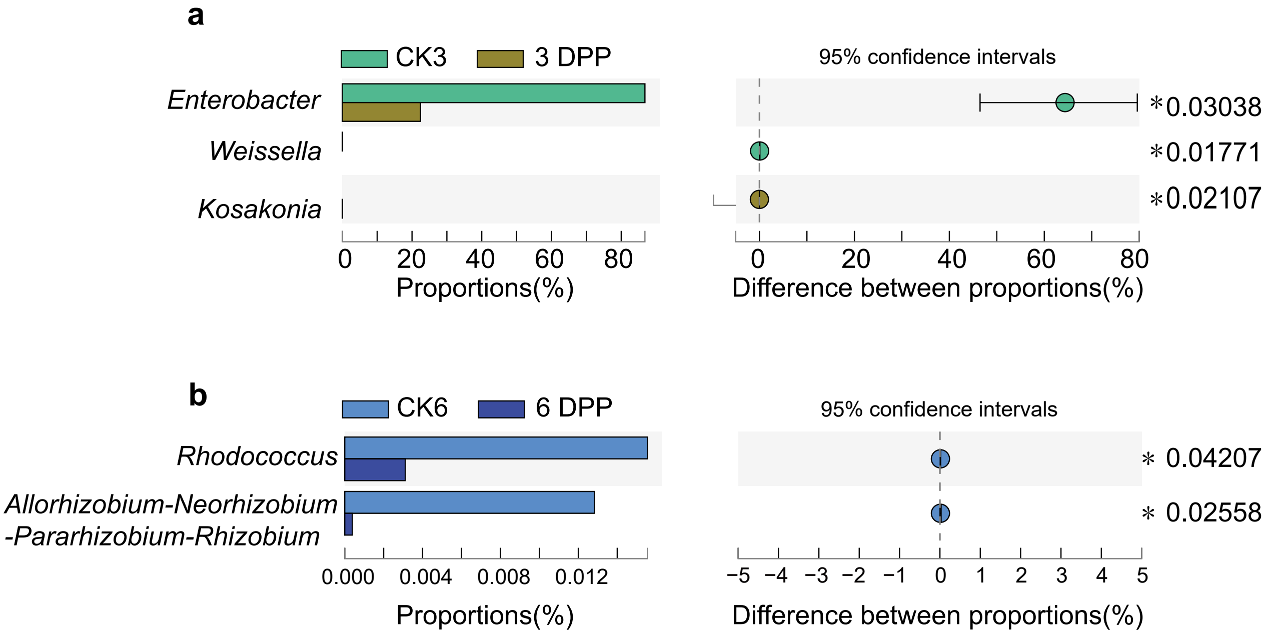

Figure S2. The difference in relative proportion(%) between parasitized and non-parasitized larvae on different sampling times at genus level. **a** CK3 vs 3 DPP, **b** CK6 vs 6 DPP. Statistical analysis was performed by the Wilcoxon rank-sum test. * $p < 0.05$.
